# Perspectives on Systems Modeling of Human Peripheral Blood Mononuclear Cells

**DOI:** 10.3389/fmolb.2017.00096

**Published:** 2018-01-09

**Authors:** Partho Sen, Esko Kemppainen, Matej Orešič

**Affiliations:** ^1^Turku Centre for Biotechnology, University of Turku and Åbo Akademi University, Turku, Finland; ^2^School of Medical Sciences, Örebro University, Örebro, Sweden

**Keywords:** systems biology, multi-omics, peripheral blood mononuclear cells, PBMCs, immune system, metabolomics, genome-scale metabolic models, pathways

## Abstract

Human peripheral blood mononuclear cells (PBMCs) are the key drivers of the immune responses. These cells undergo activation, proliferation and differentiation into various subsets. During these processes they initiate metabolic reprogramming, which is coordinated by specific gene and protein activities. PBMCs as a model system have been widely used to study metabolic and autoimmune diseases. Herein we review various omics and systems-based approaches such as transcriptomics, epigenomics, proteomics, and metabolomics as applied to PBMCs, particularly T helper subsets, that unveiled disease markers and the underlying mechanisms. We also discuss and emphasize several aspects of T cell metabolic modeling in healthy and disease states using genome-scale metabolic models.

## Introduction

Human peripheral blood mononuclear cells (PBMCs) are peripheral blood cells carrying a single round nuclei. PBMCs are comprised of several classes of immune cells, including T cells (~70%), B cells (~15%), monocytes (~5%), dendritic cells (~1%) and natural killer (NK) cells (~10%) (Autissier et al., 2010; Kleiveland, 2015). The T cell co-receptor (CD3^+^ expressing T lymphocytes) can be divided into CD4^+^ and CD8^+^ cytotoxic cells, which are present in PBMCs in approximately 2:1 ratio (Kleiveland, 2015). Activated CD4^+^ T cells are further divided into Th1, Th2, Th17, Th9, Th22, follicular helper (Tfh) cell and regulatory T cell (Treg) subsets, based on the panel of cytokines produced, transcription factors and surface markers expressed (Stockinger and Veldhoen, 2007; Sakaguchi et al., 2008; Broere et al., 2011; Crotty, 2011; Akdis et al., 2012; Luckheeram et al., 2012; Tan and Gery, 2012; Kleiveland, 2015; Golubovskaya and Wu, 2016). Treg cells can be natural cells (nTreg) generated in the thymus or inducible Treg cells (iTreg) when activated in the periphery (Wing and Sakaguchi, 2010). Likewise, activated CD8^+^ T cells (cytotoxic T cells) can be divided into Tc1 or Tc2 subsets based on their signature cytokines (Croft et al., 1994). Different subsets of T cells, their mechanisms of activation, differentiation and their functions have been extensively reviewed (Broere et al., 2011; Luckheeram et al., 2012).

B cells or B lymphocytes are bone marrow derived cells, which express the B cell receptor and bind to specific antigens against which they initiate antibody responses, thus forming the core of the adaptive humoral immune system (Cooper, 2015). B cells mature into plasmablasts and plasma cells, memory B cells, follicular B cells, marginal zone B cells, B regulatory and B-1 cells. The cytotoxic natural killer cells (NK cells), unlike T and B cells, are critical components of the innate immune system and can directly destroy pathogen infected cells. In addition, NK cells secrete lymphokines and interact with other immune cells and thus participate in immune responses by means other than direct cytotoxicity (Yuan et al., 1994).

## Systems approaches applied to PBMCs

**Figure 1:**
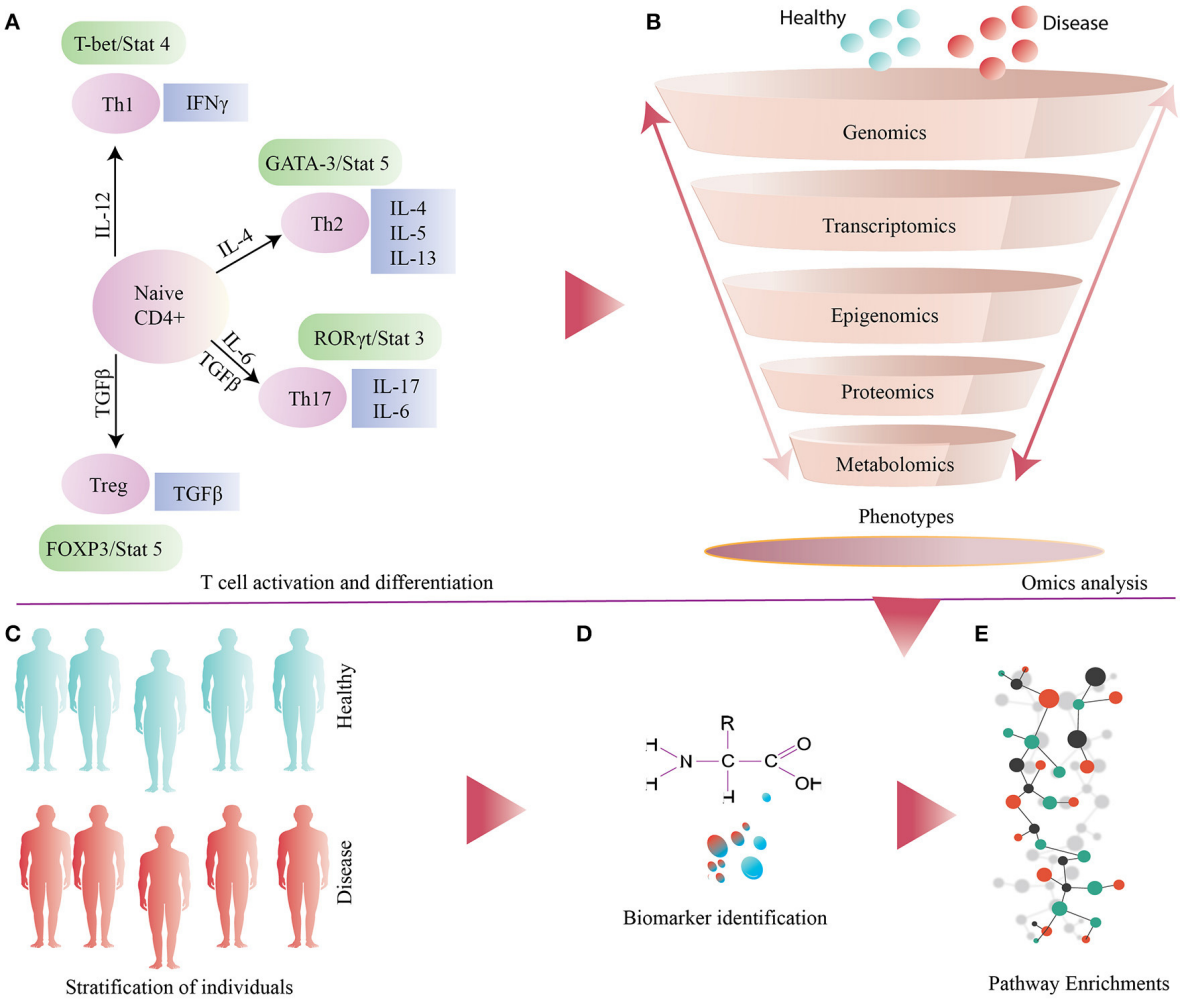
**(A)** General illustration of T cell activation and differentiation. **(B)** Several omics based approaches applied to samples obtained from disease and healthy individuals (controls). **(C)** Stratification of individuals based on metabolic phenotype. **(D)** Identification and validation of biomarkers. **(E)** Down-stream analysis of omics datasets for identification and enrichments of differential pathways.

Systems biology together with bioinformatics has begun to emerge as an essential tool in immunological research. Integration of complex multi-omics datasets has unveiled several biomarkers and elucidated their physiological role (Buonaguro et al., 2011; Li et al., 2013, 2014b, 2017b; Olafsdottir et al., 2016). PBMCs, a large complement of inflammatory cells which is easy and inexpensive to acquire, can provide a more comprehensive overview of the immune system status than circulating serum or plasma markers. PBMCs have been used extensively to study several autoimmune disorders such as type 1 diabetes mellitus (T1DM) (Foss-Freitas et al., 2008), asthma (Iikura et al., 2011; Falcai et al., 2015), numerous allergies and cancer (Payne et al., 2013). Below we provide examples of omics and systems based approaches as applied to PBMCs, particularly to T helper cells (Figure 1).

## Transcriptomics

Global transcriptomics analyses of PBMCs have been successfully used in elucidating the inflammatory mechanisms underlying different autoimmune diseases (Bennett et al., 2003; Crow et al., 2003; Greenberg et al., 2005; Achiron et al., 2007; Edwards et al., 2007). A proinflammatory transcriptional signature of interleukin-1 cytokine family was marked in patients with recent-onset of T1DM (Wang et al., 2008; Levy et al., 2012). Gene expression profiling of PBMCs using oligonucleotide array was used to identify 330 transcripts that were differentially expressed in rheumatoid arthritis (RA) patients as compared to the healthy controls (Edwards et al., 2007).

Transcriptomics data from PBMCs across multiple studies were used to characterize multiple types of diabetes, which revealed that gestational and T1DM were related at the transcriptome level (Collares et al., 2013). Meta-analysis of PBMC based microarray datasets was used to identify dysregulated pathways in patients with systemic lupus erythematosus (SLE). The study revealed that toll-like receptor (TLR) signaling, oxidative phosphorylation, diapedesis and adhesion regulatory networks were differentially regulated in the PBMCs of affected individuals (Kröger et al., 2016).

Transcriptomes from PBMCs have also been used to characterize HIV phenotypes. Distinct transcriptomics signatures with several dysregulated genes involved in apoptosis were identified in rapid HIV progressors. The expression of five miRNAs (miR-31, 200c, 526a, 99a, and 503) were also found to be altered (Zhang et al., 2013). In another study, gene expression profiling of PBMCs obtained from smokers exhibited a signature of chronic obstructive pulmonary disease (COPD) and emphysema characterized by multiple differentially regulation of genes *FOXP1, TCF7*, and *ASAH1* involved in sphingolipid (ceramide) metabolism. Plasma metabolomics validated the identity of glycoceramide as a marker of emphysema (Bahr et al., 2013).

In addition, integration of transcriptomics and protein expression profiles of PBMCs obtained from a large study cohort suggested an association between decreased IL-16 and emphysema; it also identified IL-16 cis-eQTL as a novel disease biomarker (Bowler et al., 2013). PBMCs have also been analyzed in the context of cancer. Whole genome cDNA microarray analysis study of PBMC samples from 26 patients with pancreatic cancer and 33 matched healthy controls identified an eight-gene predictor set comprising SSBP2, Ube2b-rs1, CA5B, F5, TBC1D8, ANXA3, ARG1, and ADAMTS20 (Baine et al., 2011). Similarly, significant differences were observed in the PBMC transcriptomes as obtained from renal cell carcinoma patients and normal volunteers (Twine et al., 2003; Burczynski et al., 2005).

RNA-Seq and microarray based transcriptomics datasets have been used to characterize different subsets of T helper cells. Transcriptomics of the differentiated subsets (Ciofani et al., 2012; Hu et al., 2013) characterized differences between Th17 and Th0 cells (TCR stimulated CD4^+^ T cells), while functional analysis inspired by these transcriptomes suggested differences in the control of cell cycle regulation (Simeoni et al., 2015). In another study, transcriptome analysis of cord blood-derived naïve T cell precursors was used to identify several lineage-specific genes involved in the early differentiation of Th1 and Th2 subsets (Kanduri et al., 2015). Moreover, comparative transcriptomics of mouse and human Th17 cells marked novel transcripts related to Th17 polarization. Several human long non-coding RNAs were identified in response to cytokines stimulating Th17 cell differentiation (Tuomela and Lahesmaa, 2013; Tuomela et al., 2016).

## Epigenomics

Epigenetics play a pivotal role in the regulation of gene expression and inheritance of genetic information. Epigenome-wide association studies of three human immune cell types (CD14^+^ monocytes, CD16^+^ neutrophils and naïve CD4^+^ T cells) obtained from 197 subjects were performed to assess the impact of cis-genetic and epigenetic factors. The major outcome of this study was the identification of 345 molecular trait QTLs (quantitative trait loci) which co-localized with immune disease specific loci (Chen et al., 2016). Epigenetic mechanisms in naïve CD4^+^ T cell have been extensively reviewed (Lee et al., 2006; Sanders, 2006; Aune et al., 2009; Hirahara et al., 2011; Oestreich and Weinmann, 2012).

## Proteomics

Proteome profiling of PBMCs has been carried out primarily for two purposes: (a) to identify protein biomarker(s) associated with specific pathophysiological processes, and (b) to characterize different subsets of immune cells based on their proteomes. Recently, comparative proteomics using tandem mass spectrometry (MS) was applied to PBMC samples obtained from kidney biopsies of 40 kidney allograft recipients, either with healthy transplants or those suffering acute rejection. A total of 344 proteins were identified, cataloged and mapped to 2905 proteoforms (Savaryn et al., 2016). Comparative proteome analysis also revealed differences between untreated and inflammatory activated human PBMCs (T cells and monocytes) using 2D-PAGE and LC–MS/MS. Several cell specific proteomic signatures of activation and inflammation were identified as NAMPT and PAI2 (PBMCs), IRF-4 and GBP1 (T cells), PDCD5, IL1RN, and IL1B (monocytes) (Haudek-Prinz et al., 2012).

Proteome profiling of the Th1 cells induced from naïve T cells by stimulating with interleukin 12 (IL-12) was used to identify 42 IL-12 regulated genes, among which 22 were up- and 20 were down-regulated. Functional characterization of the up-regulated proteins helped to identify a multifunctional cytokine macrophage migration inhibitory factor and a novel IL-12 target gene (Rosengren et al., 2005). In another study, MS (stable isotope labeling by amino acids in cell culture, *SILAC*) based profiling of cell surface proteome was used to identify differentially expressed proteins between human Th1 and Th2 cells. Among the differentially expressed proteins, BST2 (bone marrow stromal protein 2) and TRIM (T cell receptor interacting molecule) were found to be significantly differently regulated (Loyet et al., 2005). Moreover, global analysis of highly purified primary naïve T and Th1 cell proteomes using LC-MS/MS revealed differential regulation of ubiquitination pathway upon T cell differentiation (Pagani et al., 2015). Quantitative proteomics of Th cells using ICAT labeling and LC MS/MS have identified (557) and quantified (304) IL-4-regulated proteins from the microsomal fractions of CD4^+^ cells extracted from umbilical cord blood. Among these, small GTPases, mainly GIMAP1 and GIMAP4, were down-regulated by IL-4 during Th2 differentiation (Filén et al., 2009).

## Metabolomics

Circulating PBMCs are a complex mixture of different subsets of immune cells in highly variable stages of their lifespan. In addition to the natural genetic variation and immune challenges, this heterogeneity is shaped by the myriad of environmental conditions around them. In the light of the current understanding, the key role of the cell metabolism in immune cell function also underscores the potential impact of metabolites in regulating immune system directly or indirectly (Buck et al., 2015). For example, external perturbations to key metabolic processes such as glycolysis, energy metabolism, fatty acid and amino acid metabolism are known to affect and impair T cell activation and differentiation (Berod et al., 2014; Almeida et al., 2016; Geiger et al., 2016; Ma et al., 2017).

Metabolomics of PBMCs obtained from affected or healthy mice and humans have been used to identify metabolic markers in various pathological conditions. For example, gas chromatography coupled to MS (GC-MS) based targeted metabolomics was used to quantify glucose derived metabolites in PBMCs of healthy controls, schizophrenia and major depressions. Most of these metabolites were found to be significantly altered particularly in schizophrenic subjects. In addition, ribose 5-phosphate showed a high diagnostic performance for first-episode drug-naïve schizophrenia subjects (Liu et al., 2015). Similarly, GC–MS was used to identify metabolites such as malic acid, ornithine, L-lysine, stigmasterol, oleic acid, adenosine and N-acetyl-D-glucosamine which were significantly altered in resilient rats while statistical analysis of metabolic pathways showed aberrant energy metabolism (Li et al., 2017a).

Fatty acid composition of PBMCs phospholipids obtained from 150 subjects were estimated and linked with immune cell functions. The proportions of total polyunsaturated fatty acids (PUFAs) in PBMC phospholipids were positively correlated with phagocytosis by neutrophils and monocytes, neutrophil oxidative burst, lymphocyte proliferation, and interferon-γ production. The study also suggested that variations in the fatty acid composition of PBMCs phospholipids might induce subtle variations in immune cell functions as seen in healthy individuals (Kew et al., 2003). Since the phospholipids are primarily incorporated into cellular membranes, this effect may be mediated by the altered membrane properties such as fluidity and lateral pressure, due to their altered phospholipid composition (Mouritsen, 2011).

High-resolution MS was recently used to generate dynamic metabolome and proteome profiles of human primary naïve T cells upon activation. The study reported a dramatic decrease in intracellular L-arginine concentration which has impact on metabolic fitness and survival capacity of T cells related to anti-tumor responses (Geiger et al., 2016). Metabolism of T cells during naïve, activated, proliferative and differentiated states have been extensively reviewed (Gerriets and Rathmell, 2012; MacIver et al., 2013; Pearce and Pearce, 2013; Pearce et al., 2013; Buck et al., 2015; Dimeloe et al., 2017).

## Gut microbes and immune cells

The link between diet, gut microbiota and the immune response is currently well recognized. It is known that the immune system plays a significant role in the regulation of gut microbiota and in turn microbiota contribute to the development, training and tuning of the immune responses (Round and Mazmanian, 2009; Belkaid and Hand, 2014). Imbalances in the microbial composition or host specific interactions have been linked to inflammatory and autoimmune diseases (Brugman et al., 2006; Wen et al., 2008; Roesch et al., 2009; Kostic et al., 2015). It has been demonstrated that composition of the gut microbiota may be altered in individuals at risk of developing T1DM (Brown et al., 2011; Giongo et al., 2011; de Goffau et al., 2013; Murri et al., 2013). The phenomenon was first observed in a cohort of Finnish children at high HLA-associated risk of developing T1DM, where fecal samples from individuals seropositive with multiple pancreatic islet antigen specific autoantibodies were compared to seronegative healthy controls (Giongo et al., 2011; Kostic et al., 2015). Furthermore, Kostic et al., examined the relationship between dynamics of human gut microbiome throughout the infancy in a cohort of 33 infants genetically predisposed to T1DM. The study showed a decline in alpha-diversity in T1DM progressors between seroconversion and T1DM diagnosis; followed by an increase in microbial species which promote in inflammation, altered gene functions and stool metabolites (Kostic et al., 2015). Links between diet, gut microbiota and T cell associated disorders have been reviewed elsewhere (Kosiewicz et al., 2014; Mejía-León and Barca, 2015; Knip and Siljander, 2016).

A comprehensive list of omic approaches applied to PBMCs and T helper subsets is provided in (Table 1).

**Table 1:** List of studies performed by using PBMCs and T cells as model systems.

**Omics**	**Study**	**Cell type**	**References**	**Identifiers and other data sources**
Transcriptomics	Systemic lupus erythematosus	PBMCs	Bennett et al., 2003; Chaussabel et al., 2008; Fernandez et al., 2009; Smiljanovic et al., 2012; Kröger et al., 2016	[–, GEO: GSE11909, GSE13887, GSE38351, –]
	Dermatomyositis	PBMCs	Greenberg et al., 2005	[GEO: GSE1551]
	Acute multiple sclerosis	PBMCs	Achiron et al., 2007	–
	Rheumatoid arthritis	PBMCs	Edwards et al., 2007; Teixeira et al., 2009	[–, GEO: GSE15573]
	T1DM	PBMCs	Wang et al., 2008; Levy et al., 2012	[–, GEO: GSE35725]
	Multiple types of Diabetes	PBMCs	Collares et al., 2013	–
	HIV	PBMCs	Zhang et al., 2013	–
	COPD	PBMCs	Bahr et al., 2013	[GEO: GSE42057]
	Pancreatic cancer	PBMCs	Baine et al., 2011	–
	Renal cell carcinoma	PBMCs	Twine et al., 2003; Burczynski et al., 2005	–
	–	T cells	Ciofani et al., 2012; Hu et al., 2013	[GEO: GSE40918, GSE48138]
	–	Th1 & Th2	Kanduri et al., 2015	[GEO: GSE71646]
	–	Th17	Tuomela et al., 2016	[GEO: GSE52260]
Epigenomics	–	T cells (Naïve CD4^+^)	Tuomela and Lahesmaa, 2013; Chen et al., 2016	–
Proteomics	Kidney transplant (biopsies)	PBMCs	Savaryn et al., 2016	–
	Inflammation	PBMCs	Haudek-Prinz et al., 2012	–
	–	T cells	Filén et al., 2009	–
	–	Th1	Rosengren et al., 2005; Pagani et al., 2015	[–, PRIDE: PXD001066]
	–	Th1 & Th2	Loyet et al., 2005	–
Metabolomics	Schizophrenia and depression	PBMCs	Liu et al., 2015	–
	–	PBMCs	Kew et al., 2003	–
	–	T cells	Geiger et al., 2016; Angelin et al., 2017; Mak et al., 2017	–

*In case of meta-analysis (Kröger et al., 2016) all the datasets recruited in the study are also cited and their corresponding data identifiers and references are added. Symbol “—“ denotes no data identifier mapped to public repositories. GEO stands for Gene Expression Omnibus and PRIDE stands for PRoteomics IDEntifications database*.

## Genome-scale metabolic models as a tool to study metabolism

With the rapid advancement of cutting-edge technologies in PBMC research, there is a growing need for development of integrative methods and computational models to cope with the increasing amounts of data. These approaches when applied at the systems level could mechanistically relate entities like gene, proteins and metabolites that might unveil the disease markers and related processes at the systems level (Sen et al., 2016).

Genome-scale metabolic modeling (GSMM) is a constraint-based mathematical modeling approach that integrates biochemical, genetic and genomic informations within a computational framework (Price et al., 2004; Orth et al., 2010; Bordbar et al., 2014; O'Brien et al., 2015). It is used to study metabolic genotype-phenotype relationship of an organism. GSMM have been continuously evolving over the past 30 years. Genome-scale metabolic models (GEMs) have been used in *in silico* metabolic engineering for designing studies such as essentiality of the reaction/gene (Patil et al., 2005; Suthers et al., 2009), relevance of foreign pathway(s) (Pharkya et al., 2004) and over expression or suppression of metabolites and metabolic pathways (Pharkya and Maranas, 2006). They are efficient tools for prediction of growth in living cells/tissues exposed to different nutrients (Förster et al., 2003; O'Brien et al., 2013).

Over the past years, the components and functionalities of GEMs have been extended to study metabolism in human. The first *in silico* global reconstruction of human metabolic network Recon 1 (*1,905 genes, 3,742 reactions, and 2,766 metabolites)* was built with a vision to integrate and analyze biological datasets (Duarte et al., 2007). Subsequently, the Edinburgh Human Metabolic Network (EHMN) (*2,322 genes, 2,823 reactions, and 2,671 metabolites*) (Ma et al., 2007) was developed, these models were parsimonious and provided partial knowledge about human metabolism. Thereafter, Recon 2 (*2,194 genes, 7,440 reactions, and 5,063 metabolites*) (Thiele et al., 2013), Recon 2.2 (*1,675 genes, 7,785 reactions, and 5,324 metabolites*) (Swainston et al., 2016), a community-driven consensus human metabolic reconstruction, and Human Metabolic Reaction (HMR) (*3,668 genes, 8,181 reactions, and 9,311 metabolites*) (Mardinoglu et al., 2013, 2014) were designed that comprehensively captured human metabolism. The human metabolic reconstructions have been used to study cell, tissue and organ specific metabolism (Agren et al., 2012; Wang et al., 2012) in the context of various diseases such as cancer (Yizhak et al., 2015), non-alcoholic fatty liver disease (NAFLD) (Mardinoglu et al., 2014; Hyötyläinen et al., 2016), diabetes (Väremo et al., 2016). Furthermore, GEMs as an integrative tool has been used to model diet-tissue (Sen et al., 2017) and multi-tissue interactions in humans (Bordbar et al., 2011).

The structure of GEM provides scaffolds for integration of different types of omics data such as transcriptome, proteome and metabolome/fluxome (Blazier and Papin, 2012). Several algorithms were designed that allow integration and contextualization of GEMs based on expression datasets. GIMME designed by Becker and Palsson considers a single gene expression dataset and compares it to a certain threshold, it subsequently lists active and inactive reactions within a GEM model (Becker and Palsson, 2008). On the other hand, iMAT discretize expression dataset to low, moderate and highly expressed genes and categorize GEM reactions into low, moderate and active sets (Shlomi et al., 2008; Zur et al., 2010). MADE allows integration of multiple expression datasets, it was devised to overcome the user supplied expression threshold that might be unrealistic (Jensen and Papin, 2011). MADE decomposes gene expression data into a binary state and determines sets of low or highly active reactions. E-flux is a threshold based method that does not reduce the expression data into binary states, rather it converts the expression data to some suitable constraints that sets upper and lower limits to the reactions (Colijn et al., 2009). INIT (*Integrative Network Inference for Tissues*) algorithm uses cell specific protein abundances to generate genome-scale active metabolic networks (Agren et al., 2012).

GEMs have been used to model cataloged human gut microbes (Qin et al., 2010; Li et al., 2014a) based on their metabolic functions (El-Semman et al., 2014; Shoaie and Nielsen, 2014; Bauer et al., 2015; Magnúsdóttir et al., 2016). Magnúsdóttir et al., introduced AGORA (*Assembly of Gut Organisms through Reconstruction and Analysis*) that includes semi-automatically reconstructed GEMs of 773 human gut bacteria (205 genera, 605 species). The reconstruction can accommodate metagenomics or 16S rRNA sequencing datasets that can be used to study metabolic diversities among microbial communities (Magnúsdóttir et al., 2016). Furthermore, GEMs derived from human gut microbiome were used to decipher microbe-microbe, diet-microbe and microbe-host interactions. Another GEM based comprehensive computational platform, CASINO *(Community And Systems-level INteractive Optimization)* was designed to study the effect of diet on microbial communities (Shoaie et al., 2015).

## Genome-scale metabolic models applied to PBMCs and concluding remarks

**Figure 2:**
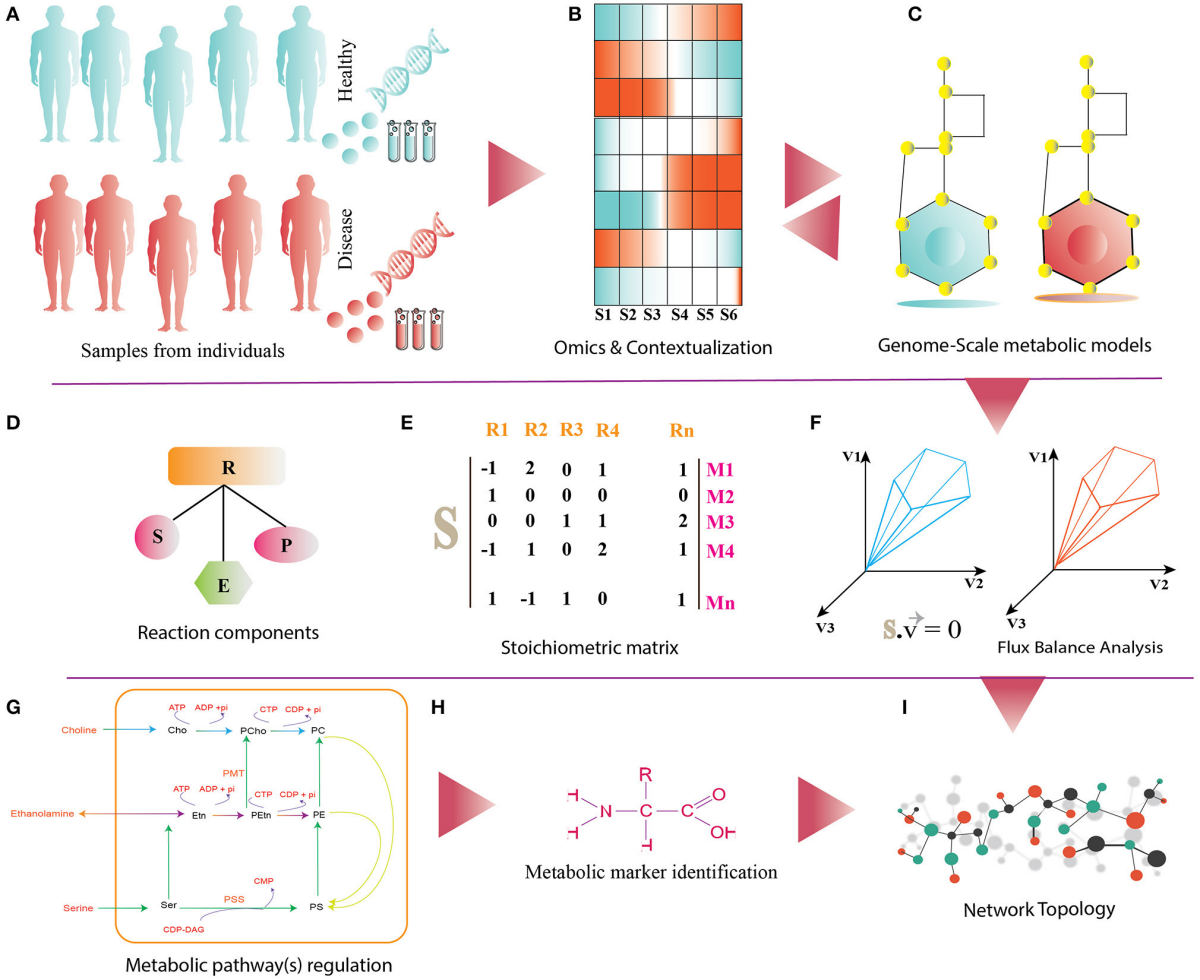
**(A)** It shows disease and healthy individuals (controls) from which PBMCs samples are obtained for omics analysis. **(B)** Differential omics expression and analysis for contextualization. **(C)** Reconstruction and contextualization of condition specific genome-scale metabolic models. **(D)** Reaction components (R) of Genome-Scale metabolic models: S, substrates; E, enzymes; P, products. **(E)** Stoichiometric matrix (S) of M_n_ metabolites and R_n_ reactions, directionality of each metabolites consumed (−1) or produced (+1) or not involved in the reaction (0). **(F)** Flux-Balance Analysis (FBA) for model simulation, optimization and estimation of flux (v) phenotype at the steady state. **(G–I)** The panel shows functionalities of genome-scale metabolic models such as regulations of metabolic pathway, metabolic marker identification and identification of differential pathways.

The availability of genome sequences of human cell lines together with the existing human metabolic reconstructions (Duarte et al., 2007; Agren et al., 2012; Wang et al., 2012; Mardinoglu et al., 2013, 2014; Thiele et al., 2013; Swainston et al., 2016; Väremo et al., 2016) and large volume of PBMC data, provides an opportunity to develop the PBMC-specific GEMs (Figure 2). These metabolic networks could be refined by the experimental data such as metabolite intensities, fluxes, enzyme abundances, and gene/transcripts expression. Network refinement adds more confidence to the metabolic reactions and their associated entities, and thus eliminates the false positives (Becker et al., 2007; Schellenberger et al., 2011). Integration of omics data with these networks makes it condition-specific, on which different analyses could be performed. One such analysis is the identification of reporter metabolites (RMs), i.e., metabolite within a metabolic network around which significant transcriptional changes occurs (Patil and Nielsen, 2005). RMs are actively involved in one or more metabolic reactions regulated by gene expression and/or enzyme abundances. RMs could also inform about the regulation of a metabolic pathway(s)/subsystem(s) (for e.g., glycolysis).

Likewise, omics data can be used to contextualize PBMC-specific networks under healthy and disease states. RM analysis can identify metabolic hotspots, modules and subnetworks, which might enhance our knowledge and understanding of immunometabolism under specific conditions. Moreover, integration of metabolomics data could help to characterize reporter reaction(s), i.e., reactions marked by significant and coordinated changes in the surrounding metabolites following the environmental/genetic perturbations. By combining transcriptome data, it is possible to infer whether the reactions are hierarchically or metabolically regulated (Cakir et al., 2006). Furthermore, fluxes estimated by PBMC-specific GEMs using Flux Balance Analysis (FBA) (Orth et al., 2010) could guide to understand the relevance of multiple pathways involved in glucose, energy, arginine and serine metabolism and ubiquinone biosynthesis with higher proficiency than previously possible (Liu et al., 2015; Almeida et al., 2016; Ma et al., 2017).

Similarly, GEMs can be reconstructed for specific immune cells. RAW 264.7 cell line, a GEM for macrophage have been developed by integration of transcriptomics, proteomics, and metabolomics datasets (Bordbar et al., 2012). The model was used to assess metabolic features that are critical for macrophage activation. It was also used to determine the metabolic modulators of the cellular activation. In another study, GEMs for naïve T cells (CD4T1670) were reconstructed by integrating transcriptomics and metabolomics datasets. This model was used to study carbohydrate metabolism, fatty acid metabolism and glutaminolysis (Han et al., 2016). Availability of the omics data for immune cell subsets, particularly CD4+ T helper cells (Th1, Th2, Th17) (Kanduri et al., 2015; Tuomela et al., 2016) provides an opportunity to reconstruct T helper specific GEMs, that could be used to characterize metabolic phenotypes of Th subsets and predict differences between them.

There is growing evidence suggesting metabolism could be regulated by epigenetic modifications (Lu and Thompson, 2012). This is facilitated by perturbation of metabolic gene(s) under suitable conditions (Colyer et al., 2012; Yun et al., 2012). Salehzadeh-Yazdi et al., incorporated epigenetic constraints in GEMs to show the impact of the mutated histone tails on metabolic reactions, thereby estimating its overall impact on yeast metabolism. The network topology was analyzed with an assumption that down-regulated metabolic genes are presumably under epigenetic control and thus affecting the metabolism of the entire organism (Salehzadeh-Yazdi et al., 2014). Similar strategy can be adopted when modeling the effect of epigenetic modification on T cell metabolism. The estimated epigenetic constraints for the down-regulated genes (presumably under epigenetic control) can be added as an additional constraint (reaction score or weight) to the associated metabolic reaction(s) within GEMs.

GEMs can be used to model and study metabolic interactions between immune cells and gut microbes on a genome-scale. This enables the identification of key regulators (metabolites/substrates, genes and enzymes) that modulate immune responses. They could also be used to identify resident microbe(s) which perform specialized metabolic functions. Moreover, GEMs can provide mechanistic overview of substrate allocation, microbe-microbe competition for resources and microbe-assisted modulation of the host immune responses. Modeling metabolic interactions among cells- and tissue-specific GEMs using a cellular compartment and/or metabolic intermediates have been previously possible (Bordbar et al., 2011; Shoaie et al., 2015; Magnúsdóttir et al., 2016; Bauer et al., 2017).

While GEMs mechanistically link metabolic genotypes and phenotypes, at the same time they handle multitude of constraints and variables which could in turn enhance uncertainty of predictions. Therefore, clear standards for GEM reconstruction, solver integration and usability has to be decided prior to the modeling (Orth et al., 2010; Chindelevitch et al., 2014; Ebrahim et al., 2015; Ravikrishnan and Raman, 2015). Availability of experimental data can help to refine GEMs to higher quality and thus lead to more accurate predictions. It is important that the predictions of GEMs are iteratively validated with the experimental data.

As indicated in this review, transcriptome, proteome, epigenome and signaling of PBMCs and Th subsets, have been well studied. In comparison, the metabolism of Th subsets and its underlying regulations is so far poorly studied. It is known that metabolism of circulating T cell undergoes dramatic changes under the environmental stress which drives the immunity (Gerriets and Rathmell, 2012; Pearce and Pearce, 2013; Pearce et al., 2013; Buck et al., 2015). We are currently making several efforts to characterize metabolic phenotype and regulations of PBMCs as obtained from pre-diabetic children at risk of developing T1DM. We believe that the congruence of GEMs based predictions and experimental data could bridge the gaps in “Big data” generated from PBMCs research. Furthermore, GEMs of PBMCs could enhance our knowledge of immune cell metabolism and allow one to better characterize PBMCs as a model system for studying immune responses under metabolically aberrant conditions.

## Author contributions

PS: drafted the manuscript; EK and MO: provided critical comments and edits to the manuscript; All authors approved the final version of the manuscript.

### Conflict of interest statement

The authors declare that the research was conducted in the absence of any commercial or financial relationships that could be construed as a potential conflict of interest.
